# The Data-Driven Definition of “Complication” in Medicine

**DOI:** 10.17691/stm2025.17.4.02

**Published:** 2025-08-29

**Authors:** G.V. Danilov, A.A. Kotov, D.Yu. Usachev, A.G. Nazarenko, Yu.V. Strunina, T.V. Tsukanova, K.B. Kotik, A.A. Potapov

**Affiliations:** MD, PhD, Head of the Laboratory of Biomedical Informatics and Artificial Intelligence; N.N. Burdenko National Medical Research Center for Neurosurgery, Ministry of Health of the Russian Federation, 16, 4^th^ Tverskaya-Yamskaya St., Moscow, 125047, Russia; PhD, Leading Research Fellow; National Research Center “Kurchatov Institute”, 1 Akademika Kurchatova Square, Moscow, 123182, Russia; Research Fellow; Russian State University for the Humanities, 6 Miusskaya Square, Moscow, 125047, Russia; Head of the Laboratory of Anthropomorphic Interfaces; Moscow State Linguistic University, 38-1 Ostozhenka St., Moscow, 119034, Russia; MD, DSc, Professor, Academician of the Russian Academy of Sciences, Director; N.N. Burdenko National Medical Research Center for Neurosurgery, Ministry of Health of the Russian Federation, 16, 4^th^ Tverskaya-Yamskaya St., Moscow, 125047, Russia; MD, DSc, Corresponding Member of the Russian Academy of Sciences, Director; Priorov Central Institution for Trauma and Orthopedics, 10 Priorova St., Moscow, 127299, Russia; Leading Engineer, Laboratory of Biomedical Informatics and Artificial Intelligence; N.N. Burdenko National Medical Research Center for Neurosurgery, Ministry of Health of the Russian Federation, 16, 4^th^ Tverskaya-Yamskaya St., Moscow, 125047, Russia; Leading Engineer, Laboratory of Biomedical Informatics and Artificial Intelligence; N.N. Burdenko National Medical Research Center for Neurosurgery, Ministry of Health of the Russian Federation, 16, 4^th^ Tverskaya-Yamskaya St., Moscow, 125047, Russia; Engineer, Laboratory of Biomedical Informatics and Artificial Intelligence; N.N. Burdenko National Medical Research Center for Neurosurgery, Ministry of Health of the Russian Federation, 16, 4^th^ Tverskaya-Yamskaya St., Moscow, 125047, Russia; MD, DSc, Professor, Academician of the Russian Academy of Sciences, Director; N.N. Burdenko National Medical Research Center for Neurosurgery, Ministry of Health of the Russian Federation, 16, 4^th^ Tverskaya-Yamskaya St., Moscow, 125047, Russia

**Keywords:** complication, definition of complication, natural language processing, word embeddings, tracking the safety of medical care, neurosurgery

## Abstract

The concept of “complication” is widely used in the medical domain to designate unfavorable events in the course of medical care. However, the medical community has not yet established a strict and generally accepted definition of “complication”. This makes it much harder to systematically track and ensure the safety of medical care, whether in an individual clinic or across the entire healthcare system.

**This study aimed** to define the concept of “complication” by identifying its generic concept and key distinguishing features using natural language processing.

**Results:**

We conducted linguistic and statistical analysis of the term “complication” using a large corpus of medical texts from 90,688 completed neurosurgical cases in the digital archive of the N.N. Burdenko National Medical Research Center for Neurosurgery, Ministry of Health of the Russian Federation, spanning 2000 to 2017. The corpus was tokenized and normalized to obtain a vocabulary of 40,121 lexemes. A total of 5853 lexemes were selected as the lexicon of adverse medical events (LAME), supposed to be found in the context of complications. Using n-gram vector representations trained on our corpus, we obtained vector representations of LAME words and selected 4416 words as the sub-LAME core based on their positive cosine similarity with the vector for “complication”. From the nouns, adjectives, and verbs in the sub-LAME, we extracted features that generalize, characterize, and classify complications. “Pathology” was identified as the generic concept for complication. The distinguishing features of complications were determined to be their novelty and emergence during observation of a primary phenomenon.

Thus, we propose the following definition of “complication” for medical care safety monitoring:

*A complication (in medicine) is an intercurrent pathology detected during observation of an underlying disease, physiological process, or the result of intervention*.

Our patented method presented in this paper enables the development of scientifically grounded definitions for unclear or poorly defined concepts.

## Introduction

The concept of “complication” in medical discourse is rather vague. Nevertheless, it is commonly used in routine practice and research without a precise definition, often with controversial interpretations. Researchers sometimes attempt to distinguish between complications, adverse events, medical errors, near-misses, consequences, and outcomes, though these terms often overlap in meaning and application across many studies [1, 2]. Paradoxically, we couldn’t find a clear and precise definition of the term “complication” in the PubMed database. Some authors discuss this term in the context of medical errors, which significantly limits its meaning. Some view complications as synonymous with adverse events. Many papers focus on defining complications related to specific pathologies within particular medical specialties. Additionally, the pharmaceutical industry provides its own more stringent definitions, but they’re limited to drug research [3].

This article explores the meaning of “complication” in the field of neurosurgery. Nevertheless, it provides a conception generalizable to other areas of medicine.

Despite complications being a crucial topic in neurosurgical research, the professional community hasn’t yet established a clear definition for the concept of “complication”. The lack of a clear definition is backed by authors in publications on neurosurgical complications [4–6]. Even though experts frequently discuss certain well-established types of complications, neurosurgeons haven’t reached a consensus on the term’s full scope of meanings [7]. This makes it impossible to compare complication rates and patterns across clinical facilities, and hampers efforts to conduct multicenter safety studies.

### Etymology of the word “complication” in English

According to Douglas Harper’s online etymological dictionary, the word “complication” has meant “a complex combination or intricate mixture” since the early XV century. It comes from the Latin word “complicatio”. This noun stems from the Latin verb “complicare”, meaning “to fold together, fold up, or roll up”. The verb itself combines two Latin elements: “com-” (meaning “together” or “with”) and “plicare” (meaning “to fold” or “weave”) [8]. Thus, at its most basic level, “complication” can be understood as “plexus”.

From the 1690s, the word took on the medical meaning of “an additional disorder which develops during the course of an existing one”, and more broadly came to signify “that which renders (an existing situation) complex, involved, or intricate” [8].

The Century Dictionary defines complication as follows: “Complication commonly implies entanglement resulting either in difficulty of comprehension or in embarrassment; complexity, the multiplicity and not easily recognized relation of parts”.

The WordNet database provides the following definitions of complication [9]:

1. [act] the act or process of complicating;
2. [state] a situation or condition that is complex or confused;
3. [state] any disease or disorder that occurs during the course of (or because of) another disease;
4. [event] ramification — a development that complicates a situation;
5. [attribute] complicatedness, knottiness, tortuousness [puzzling complexity].

The third definition above refers to the medical use of the word. In WordNet’s semantic network, “disease” serves as the hypernym (broader category) for “complication” in medical contexts. This creates a direct semantic link: complication–disease. “Complication” thus inherits the meaning of “pathological state” and appears alongside specific diseases in the classification system. For this reason, “complication” in English can function as an abstract term for any pathology or pathological condition, including standalone diseases.

In the international medical nomenclature SNOMED CT, “complication” appears in 892 concepts. Of these, 842 (94.5%) denote pathological conditions (“disorder”), while the remaining 50 concepts refer to complications of pathological or physiological conditions (such as pregnancy) or medical procedures. The breakdown includes: 19 concepts in the “situation” category, 13 in “finding”, 10 in “procedure”, 4 under “regime/therapy”, 2 in “observable entity”, and one each in “assessment scale” and “attribute” categories [10]. The isolated concept of “complication” occurs in the following hierarchy:

*I. Clinical finding: a. Disease: … i) Complication: 1) ..., 2) Adverse reaction, 3) ..., 4) Complication of procedures: (a) …, (b) Adverse event following complimentary therapy, (c) ..., 5) …; II. …*

Based on this structure, complication as a standalone concept is defined as a diagnosable pathological condition. The pathological conditions (hyponyms) listed in the Complication section number approximately 240 items. These include procedural complications and adverse reactions to medical interventions (instrumental, pharmaceutical, physical, etc.). The latter is frequently encountered in English-language scientific literature and is often used in writings about complications.

Notably, the concept “adverse event” — widely used in pharmaceutical clinical trials — appears only twice in SNOMED CT: under “assessment scale” and “disorder” (in the Complication section). This important concept thus receives insufficient attention, despite underlying many other concepts in this semantic field.

Among the general concepts for “complications”, SNOMED CT also includes “early complication”:

*1) Clinical finding: a. Clinical history and observation findings: General finding of observation of patient: General problem AND/OR complaint, Early complication …; 2) …*

**Figure 1.**
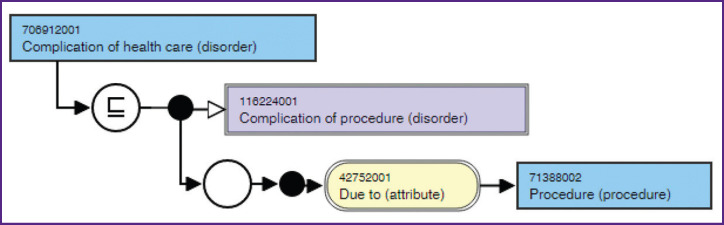
Diagram showing the semantic relationships between the concepts of “medical procedure” and “complication” in the international medical nomenclature SNOMED CT

Figure 1 illustrates the connection between the concepts of “complication” and “medical procedure” in the international medical classification system SNOMED CT.

### Etymological portrait of the word “complication” in Russian

The Russian term “complication” [*“oslozhnenie”*] closely parallels its English counterpart in many ways. The word derives from the adjective root *složn*, originally formed with the prefix *s-* and suffix *-n-* added to the root *log/lož*: *s + lož + n*. The root *log/lož* traces back to an ancient Proto-Indo-European root *legh-, which means “to lay”. Adjectival root *složn* combines with the prefix *o-* (indicating “being subjected to a state”) and the noun suffix *-enij(e)*, which creates noun from a verb root. *“Oslozhnenie”* contrasts with *“uslozhnenie”*, which uses the prefix *u-* with the same meaning of “being subjected to a state”. In modern Russian, *“uslozhnenie”* applies to most contexts meaning “getting complicated”, while *“oslozhnenie”* appears mainly in medical contexts.

According to the Russian National Corpus (https://ruscorpora.ru/), the word “complication” [*“oslozhnenie”*] first appeared in Russian during the mid-XIX century in scientific texts, serving as a direct equivalent to the modern term *“uslozhnenie*”. The first context in which the word “complication” appears in its modern sense is N. Chernyshevsky’s novel “What is to be done?” (1863). From this text, it’s evident that by the mid-XIX century, doctors were already using the word “complication” to describe features of disease progression.

During the second half of the XIX century, the term “complication” [*“oslozhnenie”*], as a “pernicious change”, began to be contrasted to the noun complication [*“uslozhnenie”*], denoting a change without negative dynamics. Interestingly, A. Chekhov used the word “complication” in his prose both to characterize a disease and to denote “pernicious changes”. Over the XX and XXI centuries, the word “complication” has continued to describe negative situations not connected to illness, though its medical meaning now dominates in modern usage.

### Research idea and objective

**Figure 2.**
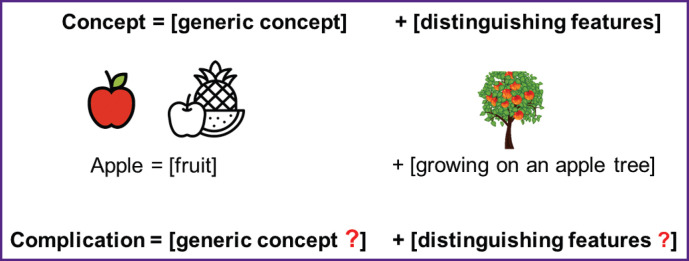
Illustration of the method for defining concepts using a formula

Linguists have a method to define concepts by using a broad category (the generic concept) and specific distinguishing features (differential features) (Figure 2). For example, an apple can be defined using the genus “fruit”, since apples belong to the fruit category. The distinguishing part of the definition should specify how an apple differs from other fruits (such as growing on an apple tree). When we apply this formula to define “complication”, the scientific task becomes finding an appropriate generic category and distinguishing features for the concept of “complication”.

Although medical ontologies and dictionaries outline some aspects of the “complication” concept, no solid scientific evidence from peer-reviewed medical literature supports a universal definition for this term. Our study aimed to define the concept of “complication” by identifying its generic category, distinguishing and classifying features through analysis of a large text corpus from electronic health records.

## Materials and Methods

To explore how the concept of “complication” is used and provide its scientifically grounded definition, we have applied a set of linguistic and computational methods for natural language processing:

1. analysis of word bi-grams containing the word “complication”;
2. analysis of contextual and semantic relationships between the word “complication” and other words using word embeddings.

### Clinical data and text corpus

The study dataset consisted of 13,060,326 text records from the electronic health records of N.N. Burdenko National Medical Research Center for Neurosurgery spanning 2000 to 2017. During this period, 90,688 neurosurgical cases were treated with 104,506 operations performed. The dataset contained de-identified (anonymized) medical narrative texts describing neurosurgical treatment cases, entered by medical personnel with physical keyboards.

These text records appeared in various sections of electronic documents, including initial exams that assessed neurological and somatic status, reports from neurologists and other specialists, surgical operation protocols, daily diary entries, results from laboratory and instrumental tests, discharge summaries, and other documentation. All these clinical texts formed the corpus for subsequent exploratory data analysis using natural language processing.

### Word bi-grams

To explore the typical usage of the word “complication”, we extracted contiguous two-word sequences (bi-grams) containing this term from the original text corpus, accounting for various morphological forms and typographical errors.

First, we preprocessed the text corpus by removing extra spaces, numbers, and all characters except Latin and Cyrillic letters. Second, we tokenized the preprocessed corpus into word bi-grams. Third, we identified unique bi-grams containing the term “complication” and related words from different parts of speech in various morphological forms (such as “*oslozhnyatsya*” (verb) meaning “getting more complicated”, “*oslozhnenniy*” (adjective) meaning “having complications”, etc.), including typographical errors. The part of speech for each word in the bi-grams was determined using MyStem software by Yandex company [11].

We used this dataset for exploratory linguistic analysis of words with the stem “*oslozhn*” (“complicat”) in Russian neurosurgical documentation. All Russian words have been literally translated in this article for presentation purposes.

### Word embeddings

We employed vector semantics to quantitatively analyze semantic relationships between terms in our text corpus [12]. Using statistics on the distribution of words and their subunits across texts, we learned word vector representations in n-dimensional vector spaces — known as word embeddings. In our preliminary research, we used various algorithms to generate 84 word embeddings, then selected those vector representations that accurately reflected semantic similarities between terms at near-expert precision. We used a benchmark set of related terms to identify the best-performing model, with details presented in our previous paper [13]. For this research, we selected character n-gram-based word embeddings (with n-grams of 3 to 6 characters). These were trained on our text corpus using cbow mode, with a window size of 5 and vector size of 200, as preliminary studies showed that this setup effectively captured the semantic closeness of words [13].

### Our approach to concept definition using vector semantics

**Figure 3.**
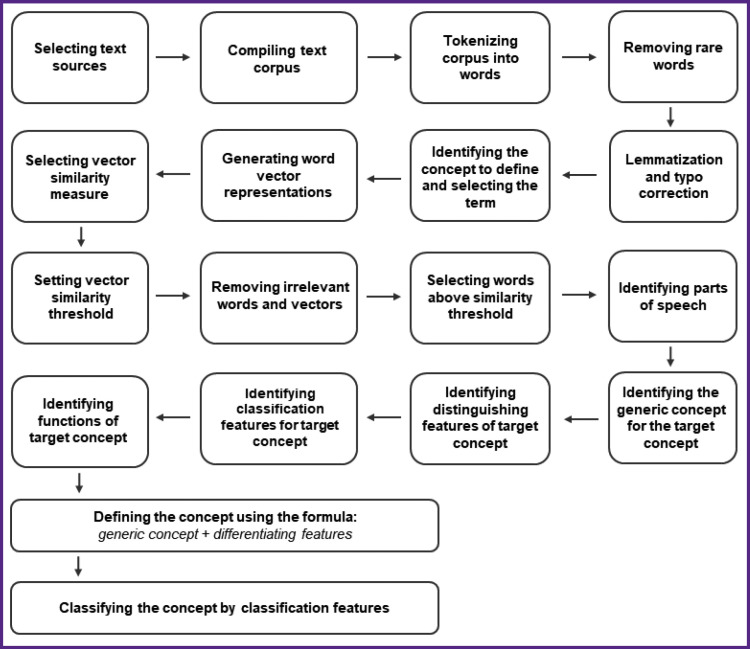
The proposed method for searching generic, differential, and classifying features to define and classify a concept (patent RU 2 795 870 C1)

In this study, we developed a methodology for deriving the core components of a concept definition through context decomposition and analysis using vector semantics (patent RU 2 795 870 C1). Figure 3 shows the scheme for the method implemented in our study. We used the text corpus described above.

We tokenized the texts of the pre-processed corpus (as described above) into individual words. We removed prepositions, conjunctions, particles, pronouns, single letters, and other stopwords. Tokens (word forms) that occurred five times or fewer in the entire corpus were also eliminated. The remaining tokens formed our main vocabulary.

Due to the morphological complexity of Russian, the specialized neurosurgical vocabulary, and the prevalence of typos in typewritten medical texts, we proceeded to lemmatize words with part-of-speech tagging (using MyStem [11]) and correct typos using an algorithm from our previous work [14]. We reduced nouns to singular masculine nominative case, adjectives to singular masculine, and verbs to infinitive form.

The resulting vocabulary contained excessive terms due to uncorrected typographical errors that lemmatization could not address. We corrected typos by clustering tokens into groups of morphologically similar ones using the Damerau–Levenshtein distance. Within each group, we treated all lemmas as morphological variants of the most common lemma (likely correctly spelled), and replaced them with it [15]. For lemmas where the corrected roots differed from the original word roots, we performed additional checks and corrections using our proprietary software. This process relied on Russian spelling rules and dictionaries. In the final vocabulary, we identified the term “complication”.

We replaced all token words in the original text corpus that appeared more than five times with their corresponding lemmas from our final dictionary. Then, we reassembled these lemmas into sequences to create a new lemmatized corpus. We used this lemmatized text set to train our vector representation model (see above) [13]. We selected cosine similarity as the measure of vector proximity, with a threshold set at zero. Higher cosine similarity values signified stronger contextual and semantic connections between words.

Since we expected “complication” to appear in contexts involving unfavorable or adverse events, we selected all word tokens that potentially denote harmful medical events (either alone or as part of phrases) in a separate list called the Lexicon of Adverse Medical Events (LAME). Next, we identified words from LAME with positive cosine similarity to “complication” in vector space, forming a subset we called sub-LAME.

We categorized each noun in sub-LAME according to its relationship with general medical concepts (symptoms, syndromes, diseases, adverse events, and synonyms or analogues of the term “complication”). This category set was developed empirically through exploratory lexicon analysis. We identified a generic category by summarizing the most frequent categories assigned to the selected nouns, considering their typical characteristics and functions. Sub-LAME adjectives were analyzed to identify differential and classificatory features of complications. We then selected verbs that indicated the functions and effects of unfavorable events, excluding those sharing common roots with previously definitely classified nouns. Finally, we used the formula “generic category + differential features” to determine the meaning of “complication” based on the decomposed context.

The data were processed, and most of the analysis was performed within the R programming environment (version 4.0.3) in RStudio Server IDE (version 1.3.1093) using tidyverse, tidytext, dplyr, Matrix, text2vec, word2vec, widyr, irlba, SnowballC, furrr, and fossil packages. Best vector representation was obtained with Python programming language (version 3.6.10) in Jupyter Notebook (version 6.1.4) using libraries to train text embedding models [13].

## Results

### Word bi-grams analysis

Tokenizing the preprocessed text corpus yielded 614,993 unique words. We identified 11,250 unique bi-grams containing the term “complication” and cognate words from a total of 282,056 bi-grams. Below, we provide a detailed overview of the findings from our exploratory bi-gram analysis.

Based on the data obtained, complications “appear”, “join”, and “develop”. In essence, a complication is identified when a new condition in the patient is detected. This pattern is evident in the following usage variants (all bi-grams translated into English; the number of corresponding bi-grams found in the corpus is indicated in brackets):

developed (development of) complications (n=259);
occurred (occurrence of) complications (n=58);
accession of complications (n=46);
appeared (appearance of) complications (n=19).

Based on these contexts, a complication is a phenomenon that is discovered in the process of the disease observation or its treatment. A broad range of initial conditions can be complicated, as illustrated by the following examples:

period [was] complicated [by] (n=3095);
operation [was] complicated [by] (n=419);
course [of the disease was] complicated [by] (n=106);
disease(s) [was (were)] complicated [by] (n=108);
treatment [was] complicated [by] (n=57);
intervention [was] complicated [by] (n=37);
condition [was] complicated [by] (n=29).

The term “complication” can denote formal causation, indicating a cause-and-effect relationship in language. For example, in these bi-grams, “complication” describes a situation “provoked” by an underlying disease or procedure:

complication of surgery (n=112);
complication of stroke (n=25);
complications of reconstruction (n=14);
complication of flu (n=13);
complication of medication (n=13);
complication of a disease (n=11);
complication of treatment (n=10);
complication of intervention (n=5).

In these phrases, the linguistic affiliation of the complication to some primary event — a disease or medical intervention — is clearly traced. This main event appears to be an integral element of the “complication” concept. At the same time, the causation reflected in language may not truly be confirmed by a causal, pathogenetic relationship between the complication and the underlying disease.

On the other hand, the word “complication” clearly appears to be a broad concept. It has a universal denotative status, that is, it indicates a large range of diseases and conditions. This is particularly evident in phrases with negation, which are the most frequent in the corpus:

without complications (58,375);
no complications (33,083);
exclude (exclusion of) complications (n=448).

The above phrases suggest that no possible situations from a set are present. Here, “complication” denotes any element within that set.

The following contexts indicate that complications are possible but not inevitable:

possible complications (5311);
risk of complications (271).

Thus, complications may accompany an underlying disease or medical procedure but are not its mandatory attribute.

### Contextual analysis of complications using vector semantics

The initial text corpus was preprocessed by removing all characters except Latin and Cyrillic letters, resulting in 229,019,413 word tokens. We then removed 159 stopwords, including prepositions, conjunctions, particles, and pronouns, from the corpus. Single-letter words in Cyrillic or Latin were also excluded, which reduced the corpus size to a sequence of 172,158,469 word forms, corresponding to 614,993 unique word forms. Word forms that appeared fewer than five times in the corpus were removed, resulting in 176,284 unique word forms. Given the relatively small proportion of unique word tokens containing Latin characters (n=8329; 4.7%), we continued the research using 167,955 unique Cyrillic word forms. Through lemmatization and typo correction, we created a vocabulary of 40,121 unique lexemes.

**Figure 4.**
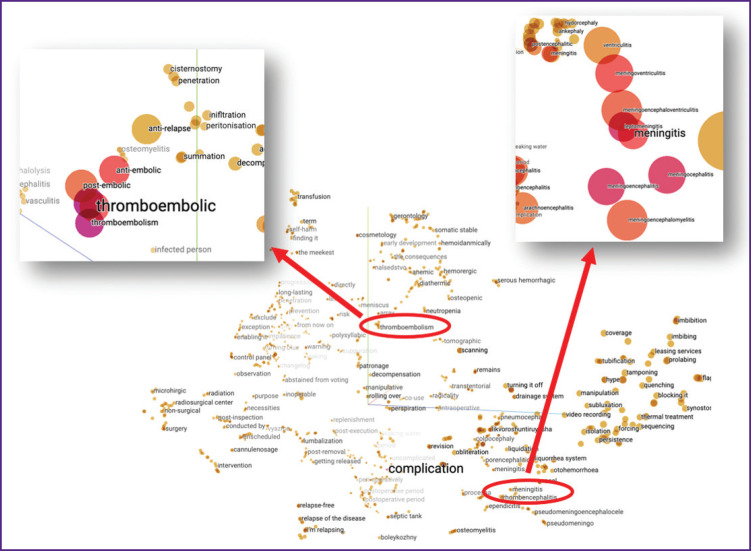
Semantic clusters of the “complication lexicon” in vector space N-gram-based word embeddings, cbow model, window size = 5, vector size = 200; 3D projection using https://projector.tensorflow.org/

We generated embeddings for each lexeme (lemma) in the vocabulary [13]. Figure 4 shows a visualization of our word embeddings projected onto three-dimensional space.

Out of 40,121 lexemes, we identified 5853 with negative connotations, likely linked to adverse medical events, in the LAME subset. We assumed that words from LAME would commonly appear in contexts involving complications. Next, we selected a sub-LAME subset from the LAME, consisting of 4416 lexemes (including 2552 nouns, 1359 adjectives, and 505 verbs) that showed positive cosine similarity (Me [Q1; Q3] — 0.141 [0.073; 0.218]) with the term “complication” in the vector space.

For each of the 2552 nouns, we identified the closest generic term (hypernym) among empirically defined categories like symptoms, syndromes, diseases, and other adverse events. The majority of selected nouns belonged to syndromes (n=1207; 47.3%), symptoms (n=729; 28.6%), and diseases (n=229; 9.0%). Additionally, 136 (5.3%) nouns represented analogs, synonyms, or metaphors for “adverse events” (such as “catastrophe” or “damage”). The remaining nouns (n=251; 9.8%) couldn’t be clearly assigned to any category without understanding the specific context of their use.

Nouns clearly categorized as symptoms, syndromes, and diseases totaled 2165 words (84.9% of all selected nouns). In WordNet’s international nomenclature, syndromes and symptoms are linked to diseases as “impairment of health or condition of abnormal functioning”. SNOMED CT nomenclature directly classifies syndromes under diseases, while both diseases and symptoms fall under clinical findings. In English and Russian medical terminology, any deviation from normal bodily function constitutes a “pathological state” or “pathological process”, consistent with the concept of “pathology” (any deviation from healthy or normal conditions).

Therefore, we suggest “pathology” — which encompasses pathological processes and conditions — as the most fitting generic concept for “complication”. Moreover, the term “pathology” remains compatible with the 136 nouns that metaphorically denote adverse events.

Next, we analyzed the 1359 selected adjectives from sub-LAME to identify features that characterize and classify complications. We defined characterizing features as adjectives that describe complications in general qualitative terms without referring to specific manifestations: unplanned, critical, unwanted, severe, unspecified, undefined, concomitant, unclear, unhappy, unpredictable, and adverse. These adjectives describe complications as newly arising and accompanying events that are unforeseen, unfavorable, undesirable, and aggravating.

Other adjectives from sub-LAME indicated specific complication characteristics: clinical manifestation (symptomatic, syndromic), underlying pathological process (ischemic, inflammatory, dyscirculatory, psychotic), localization and extent (multifocal, generalized), severity (outrageous, mild, moderate, severe, pronounced), life-threatening potential (life-threatening, lethal), temporal aspects (urgent, fulminant, emergent, recurrent), development context (nosocomial, inpatient), cause, preventability, reversibility (irreversible, reversible, unstable), and curability (incurable). We considered these adjectives as classification features of complications. Based on these features, we present complication classification principles in Appendix.

From the 505 selected sub-LAME verbs, we identified those without a common root with previously classified nouns and that indicate the functions and effects of complications. These verbs included: recur, progress, accompany, not exclude, threaten, not regress, damage, increase, warn, alter, not comply, aggravate, disable, go along with, underestimate, suspect, devitalize, harm, distort, make worse, affect, suffer, escalate, pass away, need, worry, delay, injure, burden, not subside, retard, fail, deform, toughen up, maladapt, break, die, mutilate, obstruct, not endure, compromise, deteriorate, oppress, hamper, change, perish, become more frequent, fluctuate, mutate, and exhaust. The verbs closest to “complications” in context indicated an accompanying process and its negative dynamics.

Through this analysis, we identified three critical functions of complications: (a) accompanying disease or medical intervention, (b) worsening the patient’s condition, and (c) causing harm across a wide range of manifestations.

### A data-driven definition of “complication”

Based on our comprehensive analysis, we conclude that a complication typically represents a newly detected pathology identified during observation of a disease or procedure. Complications are characterized as non-mandatory and undesirable events that accompany the primary pathology or follow a procedure. They function by worsening the patient’s condition and causing suffering through various clinical manifestations. However, since any pathology is inherently undesirable and causes patient suffering, these characteristics (being undesirable and burdensome) appear tautological and redundant in defining complications.

Analysis of bi-grams and broader contexts reveals that complications are inherently connected to medical observation processes. Complications are documented in health records alongside reports of underlying diseases or interventions. In terms of timing, they are related to the primary state or procedure. According to Grice’s maxims, the word “complication” in typical usage can lead to a naive assumption of a cause-and-effect relationship between the primary disease and the complication, however, no actual pathogenetic connection may exist.

Vector semantics identified complications as pathological entities, while bi-gram analysis demonstrated their strong association with underlying pathologies or interventions. We propose the following definition of “complication” that is suitable for monitoring safety in both neurosurgical treatment and any other types of medical care:

*Complication — an intercurrent pathology detected during observation of an underlying disease, physiological process, or the result of intervention.*

Examples of physiological processes include pregnancy. Non-therapeutic interventions such as tattooing or cosmetic procedures may also be followed by complications.

### Applying the definition to safety surveillance in neurosurgery

We believe that the proposed definition effectively meets the goals of monitoring safety in neurosurgical care, but its application can be much broader. For practical use, it’s essential to consider several key provisions.

Since complications are identified through observation, it’s essential to establish a strict time frame for monitoring. Their intercurrent nature of complications means they arise after the diagnosis of the underlying disease and before the observation period ends. Observation can start at admission (for inpatients), the first doctor’s visit, the time of intervention, or other key points. In a hospital setting, it may reasonably begin at admission and end at discharge or after a fixed interval, such as 90 days post-admission or 30 days post-surgery. These time frames should be clearly defined by specialists responsible for safety monitoring.

**Figure 5.**
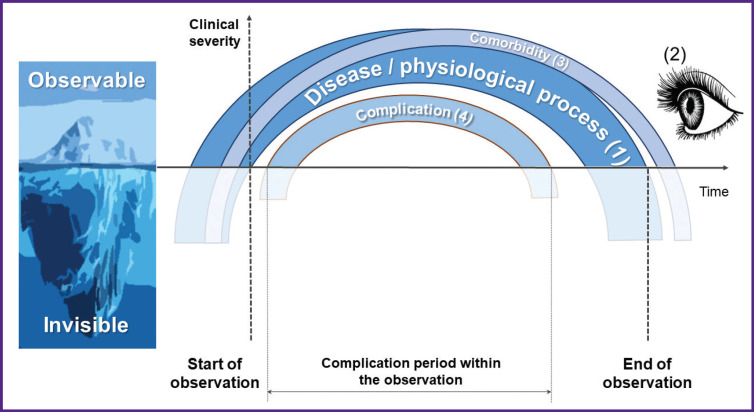
A scheme illustrating the application of the “complication” definition in clinical practice The underlying disease or physiological process (*1*) initiates external observation (*2*) that includes comorbidities (*3*) and possible manifestations of pathological processes, which may be classified as complications (*4*)

Figure 5 illustrates four key processes considered for detecting complications in clinical practice: the underlying disease or physiological process (1) that initiates external observation (2) by medical staff; possible comorbidities (3) that coexist with the underlying disease; and a complication (4) that arises only after observation starts. We recommend distinguishing between comorbidity (3) and complication (4) based on their connection to the observation process (2). A comorbidity (3) is already present and apparent when observation (2) of the underlying process (1) begins. In contrast, a complication is an intercurrent pathology discovered during the observation period and unknown at initial diagnosis. This framework sharpens the application of “complication” in standardized safety monitoring. We maintain that symptom progression documented during observation should not count as an intercurrent pathology or complication; rather, it falls under a separate adverse event category: “progression of primary symptoms”.

A clear definition of the observation period, including start and end criteria and duration, is essential for accurately detecting complications in clinical settings. The safety monitoring team must set these parameters based only on observable and objectively documented events.

Our proposed definition helps position complications within the spectrum of adverse medical events. Per this definition, a complication is a pathological condition itself, not the external factors that may cause it. For instance, under our definition, an intracerebral hematoma qualifies as a complication, whereas an accidental vascular injury during surgery that causes it does not. This view is supported by other authors, who distinguish complications from their causes [2]. It is important, that our definition steers clear of speculating on the pathophysiology or origins of complications, as these aren’t always objectively determinable in clinical practice.

## Discussion

Safety analysis in medicine faces numerous challenges. Healthcare safety improvement initiatives typically struggle with disparities between medical facilities, inadequate tools, litigation fears, heterogeneous safety models, and inconsistent conceptual frameworks. The latter is tied to the lack of a unified understanding of terminology for describing adverse events. Scientific medical literature often emphasizes grading complications without clearly defining them. Due to these issues, adverse event rates in global safety reports may be underestimated [2]. According to various studies, the overall incidence of complications in neurosurgery can be strikingly high, reaching 14% [7, 16]. However, few neurosurgical studies have attempted to develop unified complication definitions and classifications. Thus, one of the first to address the systematization of neurosurgical complications was Black in 1993 [17]. The paper presented a wide range of definitions for “complication” used across various neurosurgical institutions and questioned the feasibility of adopting universal definitions within the neurosurgical community. Lebude et al. [18] introduced a binary classification for spinal surgery complications, categorizing them as “major” or “minor”. However, this approach was overly broad. The most comprehensive classification in neurosurgery came from Landriel Ibañez et al. [19], who defined a complication as “any deviation from normal postoperative course occurring within 30 days of surgery”. This definition closely resembles that in Dindo et al.’s prominent work [20]. Nevertheless, Landriel Ibañez’s [21] classification, based on treatment methods, inadequately captured the severity of patients’ conditions. Furthermore, the definition was too vague to precisely identify complications, particularly without a standardized understanding of what a “normal” postoperative period entails in neurosurgery.

Some studies have attempted to distinguish between “complication” and “sequelae” in neurosurgery, notably the study by Likhterman and Potapov [22]. Some authors suggested attributing expected adverse surgical outcomes to treatment consequences rather than complications, which are typically unpredictable [23].

Brock et al. [6] concluded that leading neurosurgical centers worldwide should analyze their medical records to develop complexity classifications for neurosurgical operations related to risk factors and complications. We certainly agree that systematizing neurosurgical complications requires studying extensive medical record archives. Electronic health record data accumulated over time provides a good source for complications analysis [24]. Unstructured text represents the most common type of electronic medical data [25]. Natural language processing offers powerful capabilities for examining medical record content and highlighting important information for defining and testing scientific hypotheses [26]. Expert-based approaches can process textual data to define concepts and build conceptual frameworks through traditional reading [27]. However, these methods have limitations related primarily to expert subjectivity and the limited volume of text humans can process. The methodology proposed in this paper aims to overcome these limitations.

We believe a significant challenge in developing a unified, universally understood medical definition of “complications” stems from authors attempting to formulate definitions based on cause-and-effect relationships of adverse clinical events. Unfortunately, this approach, while effective when summarizing several clear cases, fails when applied to other situations. Why? The nature of adverse events and unfavorable circumstances is too complex and often unknown. If we fully understood these processes, we could easily predict and prevent them. Perhaps the intricate “interweaving” of pathological processes observed by our ancestors explains the origin of the modern word “complication” from Latin “com” + “plicare”. In our current study, we have found that conditions appearing in the same context as complications are too varied to fully grasp their “subtle” pathogenesis in every instance. Additionally, many pathologies can overlap, making it challenging to speculatively “untangle” cause-and-effect and pathogenetic relationships in clinical settings. It is worth noting that the “Good Clinical Practice” standard intentionally avoids speculating on cause-and-effect in safety monitoring, defining an “adverse event” as “an unfavourable and unintended sign (including an abnormal laboratory finding), symptom, or disease temporally associated with the use of a medicinal (investigational) product, whether or not related to the medicinal (investigational) product” [3]. The probabilistic nature of complications is aptly described by the renowned “Swiss cheese model”, which posits that complications arise from random, unpredictable events that align simultaneously [28].

Therefore, we recommend avoiding interpretations of the causes of complications and focusing solely on observable phenomena (the “tip of the iceberg”), reporting only verifiable facts. Our definition of “complication” also omits subjective terms like “undesirable”, “unfavorable”, “unnecessary”, “unintended”, and “unexpected”, as they are not practical for defining complications.

Based on our analysis, a complication is a pathology that develops and accompanies a disease or the results of medical intervention. Complications are typically discussed in terms of their “occurrence”. We emphasize the importance of recognizing their secondary nature — their necessary dependence on the underlying pathology or exposure. In essence, a complication isn’t a distinct type of pathology but a specific “role” (“function”) that pathology plays relative to the primary disease. Complications cannot exist without the context of the primary condition being observed. Thus, identifying a particular complication requires specifying the patient’s underlying condition or exposure.

Complications are noted in relation to the underlying pathology or exposures within defined time frames. We adopt this approach deliberately because linking complications to clear timelines simplifies their recording in clinical settings. Additionally, this connection helps clarify which pathology is the primary disease and which is a complication. Although the distinctions might seem instinctive, formal rules are essential to define them clearly.

How does specifying the observation time help differentiate complications from other pathological conditions, including the primary one?

First, we propose viewing the primary disease as the condition prompting medical care or observation at the time of complication detection. Formally, in outpatient and inpatient records, the main condition can be identified from the “diagnosis” section.

Our definition states that complications aren’t merely “secondary” but intercurrent, meaning they arise during another process. “Intercurrent” pathology intervenes in the primary pathology’s course, allowing identification based on observable phenomena alone, without the need for a full understanding of pathogenesis or deep causal analysis. The term “intercurrent” also signifies that no complication signs existed at observation’s onset, distinguishing complications from known comorbid conditions.

For formally confirming a pathology’s intercurrent nature (and thus its status as “a complication”), the following is required:

1. a clearly defined observation period for the underlying disease, physiological state, or intervention results;
2. absence of the pathology (complication) at the start of observation;
3. detection of the pathology (complication) exclusively during the observation period of the underlying disease, physiological state, or intervention.

An alternative to our proposed definition could be describing “complications” as any negative changes in a patient’s condition during medical observation [21]. While this makes identification easier, it obscures the distinction between the progression of an existing disease and a new pathology, and it expands the cases where a complication is diagnosed. In contrast, our definition targets a specific set of events, with their recording firmly anchored to the timeframe of medical care or observation.

We maintain that the term “adverse event”, in any context, can refer to any unwanted incident in healthcare, such as iatrogenic issues, human errors, or surgical revisions. Echoing this broad interpretation, some authors categorize adverse events to include both the emergence of pathological conditions (e.g., allergies, injuries, inflammation) and the precursors to these conditions (e.g., medication errors, surgical access mistakes, or retained foreign bodies) [29]. Unlike “adverse event”, our definition of “complication”, which specifically denotes pathology, provides greater precision. For example, leaving a foreign body in a surgical wound is an adverse event, while the subsequent inflammation from infection is the complication. Thus, complications represent a distinct subset of adverse events.

## Conclusion

Our natural language processing methodology facilitated the development of a scientifically grounded concept for defining the term “complication”. The technologies we employed enabled us to pinpoint key differential and classifying features of complications. We are confident that this methodology can be applied to clarify ambiguous concepts across various fields of human endeavor.

This study establishes a solid scientific basis for defining *a complication in medicine a*s *an intercurrent pathology detected during observation of an underlying disease, physiological process, or the result of intervention*. This definition is well-suited for monitoring medical safety. Implementing it in practice necessitates determining the observation duration, start and end criteria, a data collection protocol, and organizational procedures.

## APPENDIX

**Table:** The principles of complication classification in neurosurgery derived from unstructured text mining

Classification principles	Description
Definition	Complication of a disease Complication of a physiological condition Complication of a medical procedure Complication of a non-medical procedure
Clinical manifestation	Symptom Syndrome Disease
Organ systems	Pathology of the nervous system Pathology of the respiratory system Pathology of the digestive system Pathology of the cardiovascular system Pathology of the genitourinary system Pathology of the integumentary system Pathology of the musculoskeletal system Pathology of the immune system Pathology of the endocrine system
Conditions of observation	Nosocomial complication Community-acquired complication
Process extent	Local complication Systemic complication
Persistence	Transient (reversible) complication Persistent complication
Side of the body affected	Right-sided complication Left-sided complication
Lateralization	One-sided complication Bilateral complication
Process distribution	Focal complication Diffuse complication
Manifestation severity	Specific categories for this principle depend on the nature of the complication. Scoring tools and scales can also be used
Degree of clinical compensation	Compensated complication Subcompensated complication Decompensated complication
Onset dynamics	Complication with an acute debut Complication progressively evolving
Progression dynamics	Recurrent Sluggish Undulating
Genesis	Complication potentially associated with surgical treatment Complication potentially associated with radiotherapy Complication potentially associated with drug therapy Complication potentially related to the diagnostic procedure Complication with no apparent relationship to the underlying cause
Infectious agent presence	Infectious complication Non-infectious complication
Risk of death	Life-threatening complication Non-life threatening complication
Time of diagnosis concerning surgical treatment	Preoperative complication Intraoperative complication Postoperative complication
Prevention	Preventable Inevitable complication
Treatment potential	Curable complication Incurable complication
Treatment/observation outcome	Fatal complication Disabling complication Complication without obvious health consequences
